# Supplementary material for: Mechanical force promotes dimethylarginine dimethylaminohydrolase 1-mediated hydrolysis of the metabolite asymmetric dimethylarginine to enhance bone formation
Source: Nat Commun. 2022 Jan 10;13:50. doi: 10.1038/s41467-021-27629-2 (PMC8748781; doi:10.1038/s41467-021-27629-2)

Supplementary information for

# Mechanical force promotes dimethylarginine dimethylaminohydrolase 1-mediated hydrolysis of the metabolite asymmetric dimethylarginine to enhance bone formation

Ziang Xie[1,2,#], Lei Hou[4,#], Shuying Shen[1,2,#], Yizheng Wu[1,2], Jian Wang[5], Zhiwei Jie[1,2], Xiangde Zhao[1,2], Xiang Li[1,2], Xuyang Zhang[1,2], Junxin Chen[1,2], Wenbin Xu[1,2], Lei Ning[1,2], Qingliang Ma[1,2], Shiyu Wang[1,2], Haoming Wang[1,2], Putao Yuan[1,2], Xiangqian Fang[1,2,*], An Qin[3,*], Shunwu Fan[1,2,*]

*Corresponding authors. E-mail: orthofxq@zju.edu.cn, dr_qinan@163.com, shunwu_fan@zju.edu.cn.

**The file includes:**

Fig. S1. The concentration of ADMA in WT and Ddah2$^{-/-}$ mice.
Fig. S2. The expression of Ddah1 in BMSCs of Ddah1$^{f/f}$ and Ddah1$^{Prx1}$ mice.
Fig. S3. Exercise promoted the NO generation in bone compared with that of sedentary mice.
Fig. S4. The underlying mechanism of inhibition or activation of YAP/TAZ on the expression of DDAH1 in BMSCs or osteoblasts.
Fig. S5. The underlying mechanism of inhibition or activation of YAP/TAZ on the expression of DDAH1 in BMSCs or osteoblasts.Fig. S6. Col1ar/r mutant mice exhibit an osteopetrotic phenotype in vivo.
Fig.S6 Nuclear localization of TAZ and SMAD4 in osteoblasts under the stimulation with tension force stimulation.
Fig. S7. The effects of fluid shear stress and stiffness on osteogenesis that is regulated by TAZ/SMAD4 pathway-mediated DDAH1 expression.
Table S1. Characteristics of the individuals involved in analysis of the -394 4N del/ins polymorphism of Ddah1.
Table S2. Characteristics of the individuals involved in analysis of the serum ADMA concentrations with Spine BMD.
Table S3. Sequence of the primers used for PCR.
Table S4. Sequence of the primers used for Chromatin immunoprecipitation (ChIP) assay.
Table S5. TaqMan primer and probe sequences.

**Supplementary figure 1. The concentration of ADMA in WT and Ddah2-/- mice.**

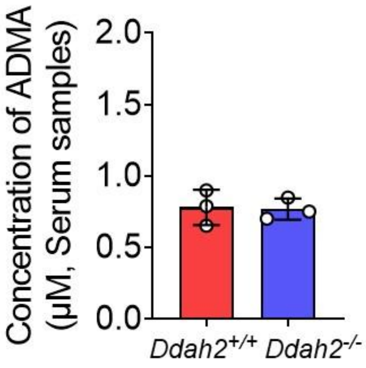


LC-MS assay to analyze the concentrations of ADMA in serum samples in *Ddah2*$^{+/+}$ and *Ddah2*$^{-/-}$ mice. *Ddah2*$^{+/+}$, n=3. *Ddah2*$^{-/-}$, n=3. No significant. Data are represented as mean values ± SD. The data were analyzed by an unpaired two-tailed Student's t-test in two groups compare.

**Supplementary figure 2. The expression of Ddah1 in BMSCs of *Ddah1$^{f/f}$* and *Ddah1$^{Prx1}$* mice.**

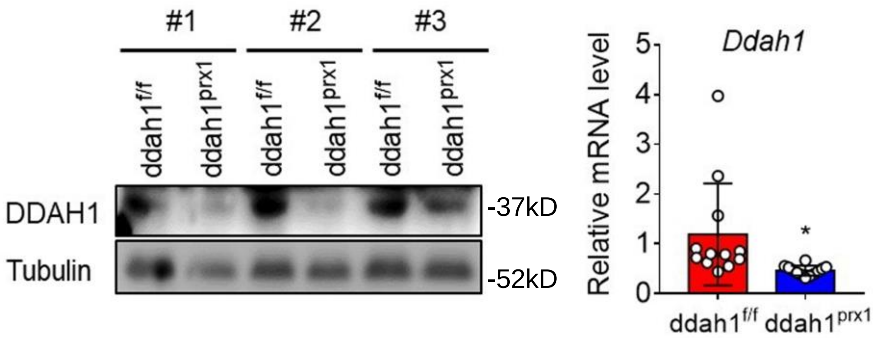


Western blot and qPCR analysis of the protein levels and mRNA levels in BMSCs of *Ddah1$^{f/f}$* and *Ddah1$^{Prx1}$* mice. *Ddah1$^{f/f}$* n=12, *Ddah1$^{Prx1}$* n=12. *, $p<0.05$. Data are represented as mean values ± SD. The data were analyzed by an unpaired two-tailed Student's t-test in two groups compare.

**Supplementary figure 3. Exercise promoted the NO generation in bone compared with that of sedentary mice.**

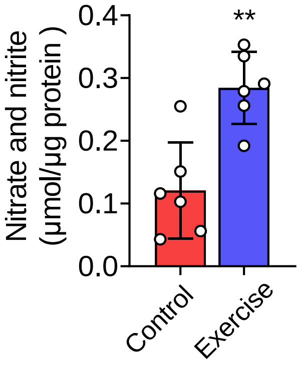


Analysis of the NO production level in all protein levels in tibiae of control and exercise treatment mice. Control, n=6. Exercise, n=6. *, $p<0.05$. Data are represented as mean values ± SD. The data were analyzed by an unpaired two-tailed Student's t-test in two groups compare.

**Supplementary figure 4. The underlying mechanism of inhibition or activation of YAP/TAZ on the expression of DDAH1 in BMSCs or osteoblasts.**

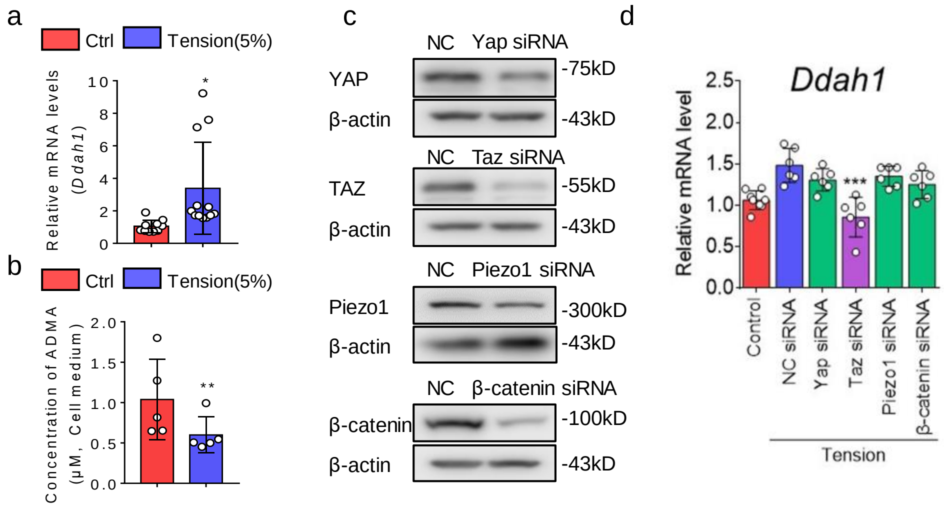


(a) The mRNA levels of Ddah1 after tension treatment. Ctrl, n=10. Tension, n=12. *, p<0.05. Data are represented as mean values ± SD. (b) ELISA assay to analyze the ADMA level of cell medium in ctrl group and tension-treatment group cells. Ctrl, n=5. Tension, n=5. **, p<0.01. Data are represented as mean values ± SD. (c) Efficiency of different siRNA knocking down in osteoblasts. (d) The mRNA levels of Ddah1 after silence of Yap, Taz, Piezao1 and β-catenin in stimulation with mechanical force. All groups, n=6. ***, p<0.005. Data are represented as mean values ± SD. The data were analyzed by an unpaired two-tailed Student's t-test in two groups compare. One-way analysis of variance (ANOVA) with post-hoc Tukey's test was used for experiments with three or more groups.

**Supplementary figure 5. The underlying mechanism of inhibition or activation of YAP/TAZ on the expression of DDAH1 in BMSCs or osteoblasts.**

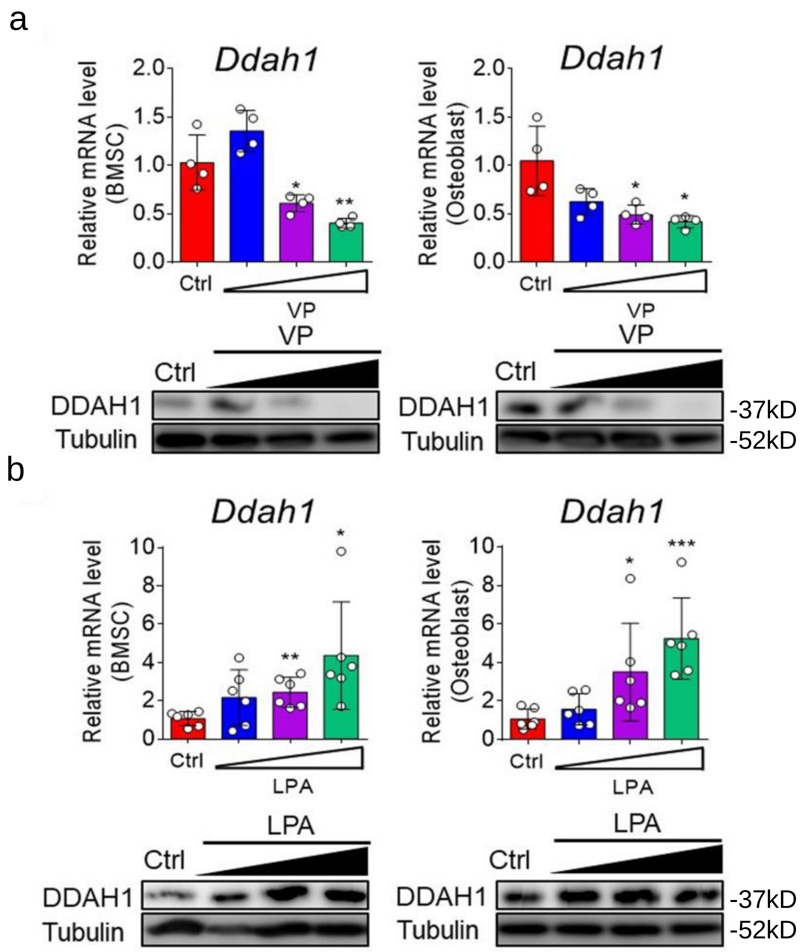


(a) The effects of a YAP/TAZ inhibitor, VP, on the mRNA and protein levels of BMSC and osteoblast with concentrations of 0.5μM, 1μM or 2μM. All groups, n=4. *, $p<0.05$. **, $p<0.01$. Data are represented as mean values ± SD. One-way analysis of variance (ANOVA) with post-hoc Tukey's test was used for experiments with three or more groups.Values represent mean ± SD. (b) The effects of a YAP/TAZ activator, LPA, on the mRNA and protein levels of BMSC and osteoblast with concentrations of 2.5μM, 5μM or 10μM. All groups, n=6. *, $p<0.05$. **, $p<0.01$. ***, $p<0.005$. Data are represented as mean values ± SD. One-way analysis of variance (ANOVA) with post-hoc Tukey's test was used for experiments with three or more groups.

**Supplementary figure 6. Nuclear localization of TAZ and SMAD4 in osteoblasts under the stimulation with tension force stimulation.**

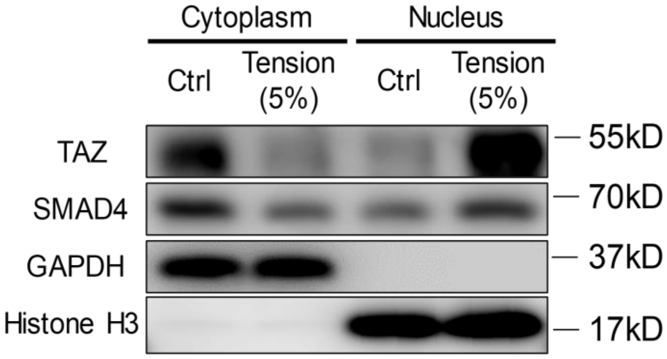

**Supplementary figure 7. The effects of fluid shear stress and stiffness on osteogenesis that is regulated by TAZ/SMAD4 pathway-mediated DDAH1 expression.**

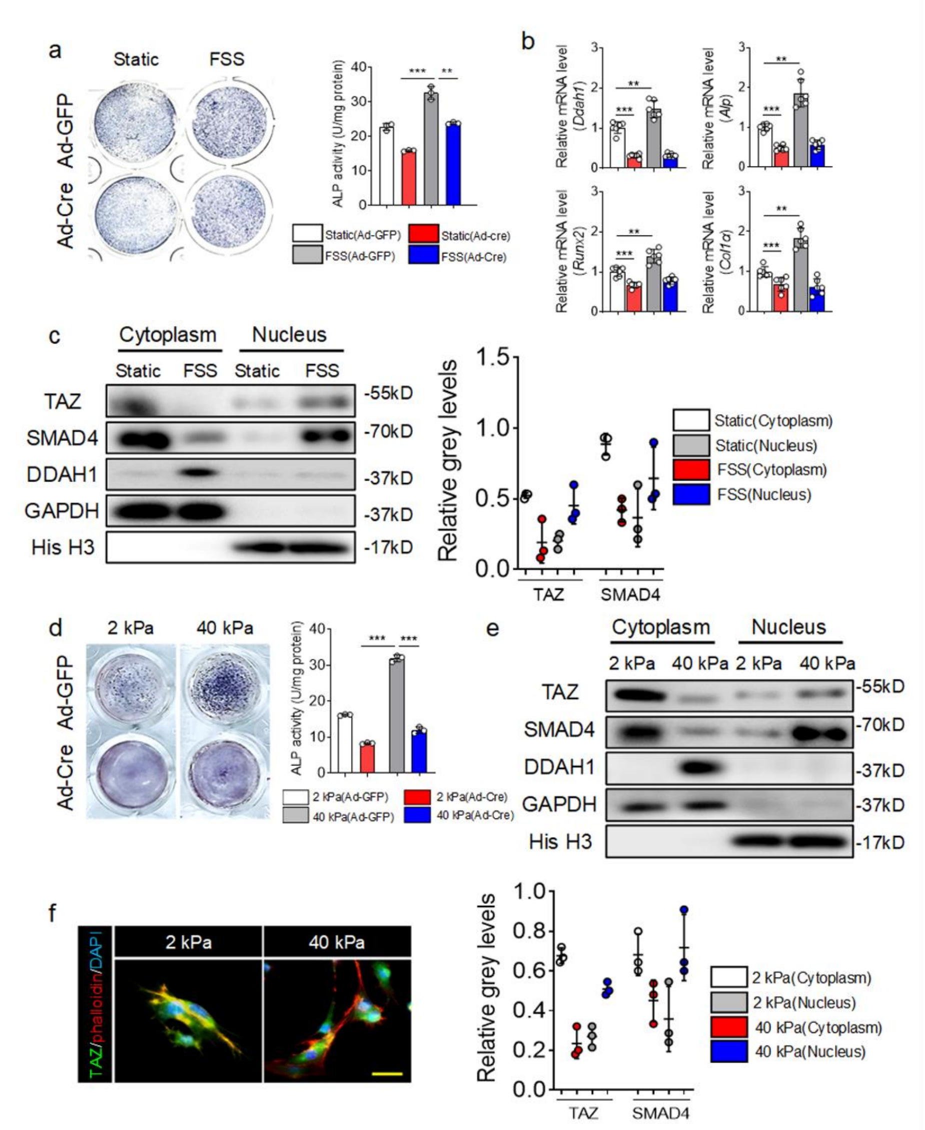


(a) ALP staining of osteoblasts from *Ddah1*$^{f/f}$ mice transfected with adenovirus-GFP or Cre after treated by fluid shear stress (FSS) or not. All groups, n=3. **, p<0.01. ***, p<0.005. Data are represented as mean values ± SD. (b) Quantitative Real-time PCR assay of osteoblasts after treated by FSS. All groups, n=6. **, p<0.01. ***, p<0.005. Data are represented as mean values ± SD. (c) Western blot assay of the indicated proteins. All groups, n=3. (d) ALP staining of osteoblasts from *Ddah1*$^{f/f}$ mice transfected with adenovirus-GFP or Cre on soft or hard stiffness GelMA matrix. All groups, n=3. **, p<0.01. ***, p<0.005. Data are represented as mean values ± SD. (e)

Western blot assay of the indicated proteins. All groups, n=3. (f) Immunofluorescence assay of the indicated proteins after cultured on soft or hard stiffness GelMA matrix. Data are represented as mean values ± SD. The data were analyzed by an unpaired two-tailed Student's t-test in two groups compare. One-way analysis of variance (ANOVA) with post-hoc Tukey's test was used for experiments with three or more groups.

**Supplementary table 1. Characteristics of the individuals involved in analysis of the -394 4N del/ins polymorphism of Ddah1.**

The study protocol was approved by the review board of Sir Run Run Shaw Hospital, and informed consent was obtained from all participants. Samples were recruited between 2019 and 2020 from 2 hospitals in Hangzhou of China. Confirmation of BMD was based on the results of T values according to World Health Organization (WHO) criteria. Patients diagnosed with severe systemic diseases such as pulmonary fibrosis, endocrine and metabolic disease, severe inflammatory diseases, autoimmune diseases, tumors and serious chronic diseases (e.g. hepatic cirrhosis, renal failure), were excluded from the study.

| Samples (n=1404) | del/del (n=1061) | del/ins (n=305) | ins/ins (n=38) |
|---|---|---|---|
| Age, years | 64.13±0.29 | 63.89±0.52 | 66.29±1.62 |
| Women, n(%) | 756(71.25) | 232(76.07) | 25(65.79) |
| Men, n(%) | 305(28.75) | 73(23.93) | 13(34.21) |
| BMI, $kg/m^2$ | 23.75±0.11 | 23.84±0.19 | 22.72±0.47 |
| Spine BMD, $g/cm^3$ | 1.23±0.01 | 1.03±0.01 | 0.96±0.03 |
| Femoral BMD, $g/cm^3$ | 0.82±0.00 | 0.80±0.01 | 0.79±0.03 |

**Supplementary table 2. Characteristics of the individuals involved in analysis of the serum ADMA concentrations with Spine BMD.**

| Samples (n=570) | Control (n=190) | Osteopenia (n=117) | Osteoporosis (n=263) |
|---|---|---|---|
| Age, years | 60.55±0.97 | 64.00±1.12 | 69.92±0.59 |
| Women, n(%) | 84(44.21) | 78(66.67) | 220(83.65) |
| Men, n(%) | 106(55.79) | 39(33.33) | 43(16.35) |
| BMI, $kg/m^2$ | 24.16±0.29 | 23.33±0.41 | 21.67±0.26 |
| Spine BMD, $g/cm^3$ | 1.21±0.01 | 0.94±0.01 | 0.74±0.01 |

**Supplementary table 3. Sequence of the primers used for PCR.**

| Genes | Upstream (5′-3′) | Downstream (5′-3′) |
|---|---|---|
| ***Gapdh(mouse)*** | *ACCCTTAAGAGGGATGCTGC* | *ATCCGTTCACACCGACCTTC* |
| ***Alp(mouse)*** | *ATCTTTGGTCTGGCTCCCATG* | *TGAGCGACACGGACAAGAAGCCCTT* |
| ***Runx2(mouse)*** | *TTACCTACACCCCGCCAGTC* | *TGCTGGTCTGGAAGGGTCC* |
| ***Col1α1(mouse)*** | *GACGCCATCAAGGTCTACTG* | *ACGGGAATCCATCGGTCA* |
| ***Bglap(mouse)*** | *GTATGGCTTGAAGACCGCCT* | *GACAGGGAGGATCAAGTCCC* |
| ***Ddah1(mouse)*** | *GCGAGGAGGTGGATTTCGCT* | *GTCCTCCACGAACACGCAGT* |
| ***Ddah1(homo)*** | *AACCACATTTCTGACACATCTTTG* | *GTAGCACAGTGGCACAGTAGATTG* |
| ***Gapdh(homo)*** | *TGGAAGGACTCATGACCACAGT* | *GCCATCACGCCACAGTTTC* |

**Supplementary table 4. Sequence of the primers used for Chromatin immunoprecipitation (ChIP) assay.**

| **Names** | **Upstream (5′-3′)** | **Downstream (5′-3′)** |
|---|---|---|
| **Site1** | *CTAAGGTCACAGTCAAATCAT* | *AGTTCAGTAGAGGATACATAAGA* |
| **Site2** | *TTCTTATGTATCCTCTACTGAACT* | *TAATTGAACGAGGTGTGGT* |
| **Site3** | *CGAAGGTCA AGGAACTGA* | *ATGTGATTCCAATGTGTTTATTTC* |
| **Site4** | *CTGGATGAGGTGGTTGAT* | *TGAAGGCACAGAG ATAGG* |
| **Site5** | *ATCCCTCACCCTATCTCT* | *GCAC TAGGCTTGTCATTC* |
| **Site6** | *AGGCATTCTTCCCACTTTA* | *TTCTCCAAATTGACCAACTG* |
| **Site7** | *CGAGGTAATTTGGGTGAAG* | *GTGAAGGGCGATTGTTTA* |

**Supplementary table 5. TaqMan primer and probe sequences.**

| | Primer sequences (5'-3') | Allele | Probes |
|---|---|---|---|
| Forward | CAGGTAAAGACCAGGAAGCCC | -394 4N del | FAM-CGCAGGTGCACACCTCCCATC-BHQ1 |
| Reverse | GGACCTCGGCGAAAAGC | -394 4N ins | HEX-CGCAGGTGCACGCACACCTC-BHQ1 |

Uncropped blots for figure 3 and figure 5.

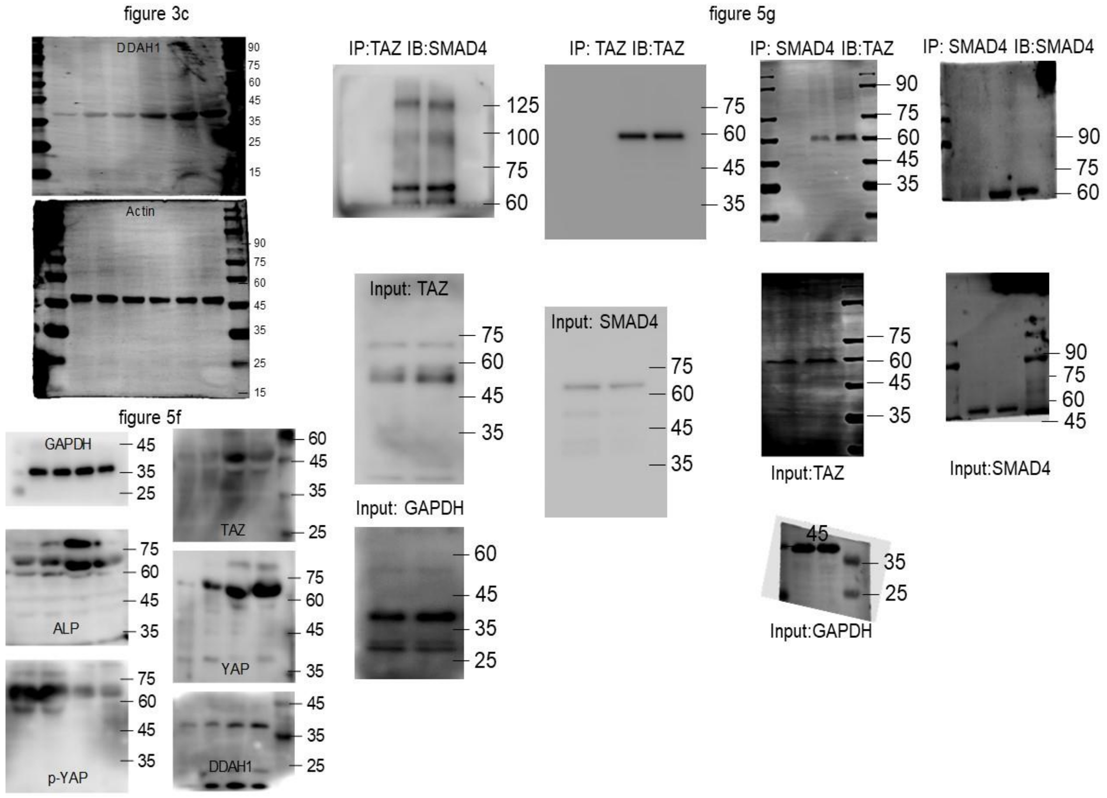


Uncropped blots for supplementary figures..

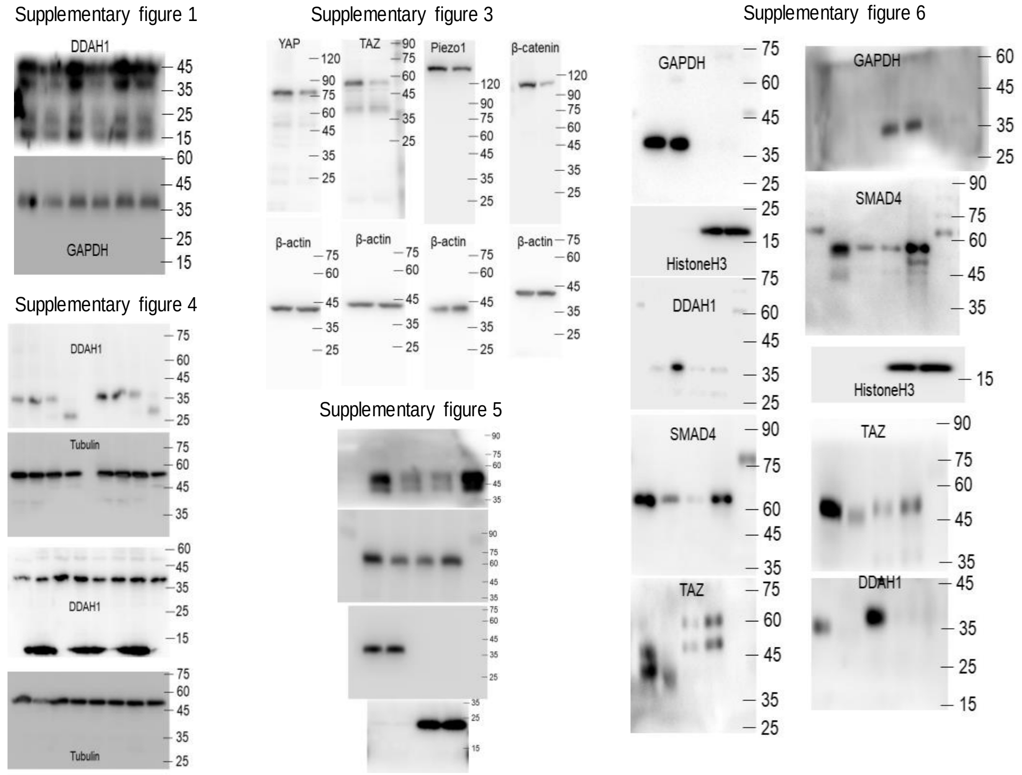

Uncropped gels for Fig. 3

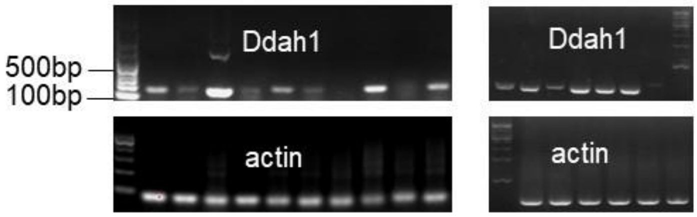


Uncropped gels for Fig. 5

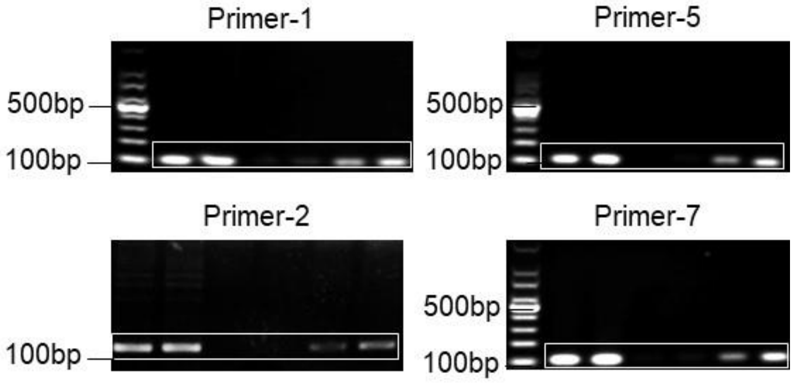

Supplement: Supplementary file 1 — Supplementary information [file 41467_2021_27629_MOESM1_ESM.pdf]
