# Supplementary material for: Mechanical force promotes dimethylarginine dimethylaminohydrolase 1-mediated hydrolysis of the metabolite asymmetric dimethylarginine to enhance bone formation
Source: Nat Commun. 2022 Jan 10;13:50. doi: 10.1038/s41467-021-27629-2 (PMC8748781; doi:10.1038/s41467-021-27629-2)
Supplement: Supplementary file 3 — Reporting Summary [file 41467_2021_27629_MOESM3_ESM.pdf]

nature portfolio

Corresponding author(s): Shunwu Fan, An Qin, Xiangqian Fang

Last updated by author(s): Sep 13, 2021

Reporting Summary

Nature Portfolio wishes to improve the reproducibility of the work that we publish. This form provides structure for consistency and transparency in reporting. For further information on Nature Portfolio policies, see our Editorial Policies and the Editorial Policy Checklist.

Statistics

For all statistical analyses, confirm that the following items are present in the figure legend, table legend, main text, or Methods section.

n/a	Confirmed	
☐	☒	The exact sample size (*n*) for each experimental group/condition, given as a discrete number and unit of measurement
☐	☒	A statement on whether measurements were taken from distinct samples or whether the same sample was measured repeatedly
☐	☒	The statistical test(s) used AND whether they are one- or two-sided<br>*Only common tests should be described solely by name; describe more complex techniques in the Methods section.*
☒	☐	A description of all covariates tested
☒	☐	A description of any assumptions or corrections, such as tests of normality and adjustment for multiple comparisons
☐	☒	A full description of the statistical parameters including central tendency (e.g. means) or other basic estimates (e.g. regression coefficient) AND variation (e.g. standard deviation) or associated estimates of uncertainty (e.g. confidence intervals)
☐	☒	For null hypothesis testing, the test statistic (e.g. *F*, *t*, *r*) with confidence intervals, effect sizes, degrees of freedom and *P* value noted<br>*Give P values as exact values whenever suitable.*
☒	☐	For Bayesian analysis, information on the choice of priors and Markov chain Monte Carlo settings
☒	☐	For hierarchical and complex designs, identification of the appropriate level for tests and full reporting of outcomes
☒	☐	Estimates of effect sizes (e.g. Cohen's *d*, Pearson's *r*), indicating how they were calculated

Our web collection on statistics for biologists contains articles on many of the points above.

Software and code

Policy information about availability of computer code

Data collection	The micro-CT data were collected using a μ-QCT system SkyScan1176 (Bruker, Kartuizersweg, Belgium). Immunofluorescence images were collected by using Olympus IX81 confocal microscope or Zeiss LSM-880 confocal microscope. The PCR data were collected by using LightCycler 480 real-time PCR system (Roche Life Science, China). Luciferase activity was measured using a luciferase assay system (Promega, USA).
Data analysis	The micro-CT image data were reconstructed and analyzed using Mimics software (v.13.0, Materialise NV). The bone statistical histomorphometric analyses were performed using the Osteomeasure software (Osteometrics, USA). The osteoclast resorption area per well was analyzed by image analysis freeware ImageJ (National Institutes of Health, USA). The protein density was quantified using freeware ImageJ (National Institutes of Health, USA). The acquired LC-MS raw data were analyzed by the progenesis QI software (Waters Corporation, Milford, USA). Graphs and statistics were prepared using GraphPad Prism 8.0 software.

For manuscripts utilizing custom algorithms or software that are central to the research but not yet described in published literature, software must be made available to editors and reviewers. We strongly encourage code deposition in a community repository (e.g. GitHub). See the Nature Portfolio guidelines for submitting code & software for further information.

Data

Policy information about availability of data

All manuscripts must include a data availability statement. This statement should provide the following information, where applicable:

- Accession codes, unique identifiers, or web links for publicly available datasets
- A description of any restrictions on data availability
- For clinical datasets or third party data, please ensure that the statement adheres to our policy

The authors declare that the data supporting the findings of this study are available within the paper and its Supplementary information files. Any remaining data

that support the results of the study will be available from the corresponding author upon reasonable request. A reporting summary for this article is available as Supplementary Information file. Source data are provided with this paper.

Field-specific reporting

Please select the one below that is the best fit for your research. If you are not sure, read the appropriate sections before making your selection.

☒ Life sciences ☐ Behavioural & social sciences ☐ Ecological, evolutionary & environmental sciences

For a reference copy of the document with all sections, see nature.com/documents/nr-reporting-summary-flat.pdf

Life sciences study design

All studies must disclose on these points even when the disclosure is negative.

Sample size	No statistical analysis was used to predetermine samples sizes. The samples sizes in this study were sufficient for analyzing and conducted the conclusion. For in vitro experiments, 3 biological replicates were used for experiments. For animal experiments, the number of mice were described in the text.
Data exclusions	Undetecable data and drastic outliers due to technical variability data were excluded.
Replication	The volume of samples for LC-MS assay was only enough for once testing, thus no replicates were performed for LC-MS. Other experiments in this study were independently repeated for three times, and the number of repicates for each experiment were presented in the text. We confirm that all attempts at replication were successful.
Randomization	For animal samples, all samples were randomly numbered and deallocated into separate groups, as well as for in vitro experiments. In brief, cells were plated randomly and plates were randomly assigned to experimental or control groups.
Blinding	Investigators in this study are blinded to groups of mice samples from micro-CT and histological analysis. For in vitro experiments (gene transfection, drug treatment, gel loading for western blot) the experiments were not blinded to allocation because these experiments required that the investigators were not blinded, so as to show the data in all figures with appropriate order. However, the investigator who perform measurements was not informed about the goals of the study and the nature of the treatment groups.

Reporting for specific materials, systems and methods

We require information from authors about some types of materials, experimental systems and methods used in many studies. Here, indicate whether each material, system or method listed is relevant to your study. If you are not sure if a list item applies to your research, read the appropriate section before selecting a response.

Materials & experimental systems

n/a	Involved in the study	
☐	☒	Antibodies
☒	☐	Eukaryotic cell lines
☒	☐	Palaeontology and archaeology
☐	☒	Animals and other organisms
☐	☒	Human research participants
☒	☐	Clinical data
☒	☐	Dual use research of concern

Methods

n/a	Involved in the study	
☒	☐	ChIP-seq
☒	☐	Flow cytometry
☒	☐	MRI-based neuroimaging

Antibodies

Antibodies used	mouse-anti-osteocalcin (Santa Cruz, 1:100, sc-376726), mouse-anti-Ddah1 (Santa Cruz, 1:100, sc-271337), rabbit-anti-Ddah2 (Abcam, 1:200, ab232694), rabbit-anti-TAZ (Abcam, 1:200, ab242313), rabbit-anti-YAP (Abcam, 1:200, ab52771), eNOS (Santa Curz, 1:200, sc-376751). Goat-anti-mouse FITC (1:1000; Jackson ImmunoResearch, 705-165-147) and donkey-anti-rabbit Alexa Fluor 488 (1:1000; Molecular Probes, A21206) were used as secondary antibodies. DAPI (Cell Signaling Technology, #4083) and DyLight™ 594 Phalloidin (Cell Signaling Technology, #12877) . DDAH1 (1:1,000; SAB, #37368), phospho-YAP (1:1,000; Cell Signaling Technology, #4911), YAP (1:1,000; Cell Signaling Technology, #14074), TAZ (1:1,000; Cell Signaling Technology, #83669), ALP (1:500; Santa Cruz, #sc-271431), SMAD4 (1:1,000; Cell Signaling Technology, #46535), GAPDH (1:5,000; Proteintech, #60004-1-Ig), alpha-tubulin (1:5,000; Proteintech, #66031-1-Ig), Histone H3 (1:5,000; Proteintech, #17168-1-AP).
Validation	Mouse-anti-osteocalcin(Mouse; IF,WB,IP,ELISA), rabbit-anti-Ddah1(Human, Mouse, Rat; WB,IHC), mouse-anti-Ddah1 (Mouse, Rat, Human; IF,WB,IP,ELISA), rabbit-anti-Ddah2 (Mouse, Rat, Human; IF,IHC,WB), mouse-anti-TAZ (Human, Mouse; WB,IHC,IF), rabbit-anti-YAP (Mouse, Human;IF,IHC,WB), mouse-anti-eNOS (Mouse, Rat, Human; IF,WB,IP,IHC,ELISA). Rabbit-anti-phospho-YAP (Human, Mouse, Rat; WB), Rabbit-anti-YAP (Human, Mouse, Rat, Monkey; WB), Rabbit-anti-TAZ (Human, Mouse, Rat, Monkey; WB,IP,CHIP), ALP (Human, Mouse, Rat; WB,IHC,ELISA), rabbit-anti-SMAD4 (Human, Mouse, Rat, Monkey; WB,IP,CHIP), mouse-anti-GAPDH

(Human, Mouse, Rat, Monkey; WB,IP,IHC,IF), mouse-anti-alpha-tubulin (Human, Mouse, Rat, Monkey, Chicken, Goat; WB,IP,IHC,IF), rabbit-anti-Histone H3 (human, mouse, rat; ChIP, IF, WB).

Animals and other organisms

Policy information about studies involving animals; ARRIVE guidelines recommended for reporting animal research

Laboratory animals	Generation of Ddah1-/- mice were described previously, a gift from Professor Yingjie Chen, Minnesota University [27]. Mice with Ddah1 conditionally knocked out (Ddah1 cKO) in osteoblast lineage cells were generated by crossing 12-week old male Ddah1f/f mice (a gift from Professor Yingjie Chen, Minnesota University) with 12-week-old female Prx1-cre mice (Jackson Lab #005584). Male Ddah1f/f and Ddah1Prx1 mice were sacrificed at 12 weeks of age. Male and female Ddah2+/- mice (C57BL/6N-Ddah2tm1cyagen) were offered by Cyagen Biology Technology. 6-week-old Female Wild type C57BL/6 (B6) mice were from the SLAC Laboratory Animal Company (Shanghai, China). The mice were housed with conditions of 12 hours dark/12 hours light cycle, 22°C ambient temperature, and 50% humidity.
Wild animals	This study did not involve wild animals.
Field-collected samples	This study did not involve field-collected samples.
Ethics oversight	All animal studies were performed according to approved guidelines for the use and care of live animals (Guideline on Administration of Laboratory Animals released in 1988, and 2006 Guideline on Humane Treatment of Laboratory Animals from China). All of the experimental procedures were approved by the Committees of Animal Ethics and Experimental Safety of Sir Run Run Shaw Hospital, Zhejiang University.

Note that full information on the approval of the study protocol must also be provided in the manuscript.

Human research participants

Policy information about studies involving human research participants

Population characteristics	Over 50-year old postmenopausal women and over 50-year old men were involved in this study. Samples were recruited between 2019 and 2020 from 2 hospitals in Hangzhou of China. Confirmation of BMD was based on the results of T values according to World Health Organization (WHO) criteria. Patients diagnosed with severe systemic diseases such as pulmonary fibrosis, endocrine and metabolic disease, severe inflammatory diseases, autoimmune diseases, tumors and serious chronic diseases (e.g. hepatic cirrhosis, renal failure), were excluded from the study.
Recruitment	Patients diagnosed with severe systemic diseases such as pulmonary fibrosis, endocrine and metabolic disease, severe inflammatory diseases, autoimmune diseases, tumors and serious chronic diseases (e.g. hepatic cirrhosis, renal failure), were excluded from the study. The exercise ability, smoking, drinking, age, BMI or sex might be impact the results in the study.
Ethics oversight	The human study was approved by the Medical Ethics Committees of Sir Run Run Shaw Hospital.

Note that full information on the approval of the study protocol must also be provided in the manuscript.
